# An experimental study of early cardiovascular disease and risk factors in a collagen-induced arthritis rat model

**DOI:** 10.3389/fimmu.2026.1801144

**Published:** 2026-04-21

**Authors:** Chun Yun Jiang, Da Li, Quan Jiang, Fengtao Pang, Run Yuan

**Affiliations:** Department of Rheumatology, Guang’anmen Hospital, China Academy of Chinese Medical Sciences, Beijing, China

**Keywords:** cardiovascular disease, collagen-induced arthritis, diastolic dysfunction, myocardial fibrosis, rat model, rheumatoid arthritis, risk factors

## Abstract

**Introduction:**

This study investigated the pathological characteristics and risk factors of early cardiovascular disease (CVD) associated with rheumatoid arthritis (RA) using a collagen-induced arthritis (CIA) rat model.

**Methods:**

A total of 120 SPF-grade male SD rats were used. Following CIA induction, echocardiography, histopathology, molecular biology, and lipid profile analysis were employed to dynamically monitor cardiac function, structural changes, and lipid profiles across disease stages (D0-D112) .

**Results:**

Sequential progression of cardiac dysfunction: Echocardiography revealed a significant decrease in the mitral valve E/A ratio (MVE/A) starting from D84 (P<0.05), indicating diastolic impairment preceding systolic dysfunction.Myocardial structural abnormalities: The heart-to-body weight ratio (HW/BW) in CIA rats was significantly higher than that in the control group starting from day 84 (*P < 0.01), indicating cardiac hypertrophy. Masson staining revealed progressive myocardial fibrosis with increasing collagen deposition from D56. Abnormal lipid profiles and biomarkers: LDL-C significantly increased from D84 to D112 (*P<0.01), while OX-LDL rose from D98 to D112 (P<0.05); B-type natriuretic peptide (BNP) significantly increased at D112 (*P<0.01), indicating heart failure risk. Negative atherosclerosis findings: No plaque formation was detected in the Oil Red O staining of the aorta and coronary arteries. While macroscopic atherosclerotic plaques were not apparent, microvascular endothelial dysfunction may play a role in the early stages of cardiovascular disease (CVD) .

**Discussion:**

Cardiovascular lesions in CIA rats exhibit sequential progression: diastolic dysfunction and myocardial fibrosis emerge first (from D56), followed by systolic dysfunction (from D98), accompanied by lipid metabolism disorders driven by LDL-C and OX-LDL. This model provides an experimental basis for studying the mechanisms of RA-associated cardiac lesions and early intervention.

## 1 Introduction

Rheumatoid arthritis (RA) is the most prevalent systemic autoimmune disease encountered in clinical practice. It causes damage to the synovial membrane, cartilage, and bones, and can also impact various internal organs, including the heart, lungs, kidneys, and digestive tract. Similar to other inflammatory joint diseases, RA is linked to an elevated risk of cardiovascular disease (CVD) (1). Clinical studies indicate that the incidence of CVD among RA patients ranges from 30% to 60%, primarily encompassing heart failure, coronary heart disease (CHD), ischemic heart disease, pericarditis, myocarditis, arrhythmias, and valvular disease. The mechanisms underlying cardiac disease in RA remain incompletely understood (2). Endothelial dysfunction in the coronary arteries, which may not necessarily correlate with traditional cardiovascular risk factors (3) or disease activity (4), could serve as a primary contributor to CHD in RA. More than 50% of RA patients without overt signs of CVD exhibit left ventricular systolic and diastolic dysfunction (5), which may be associated with the progression of heart failure in RA (6).

In this context, preclinical studies utilizing animal models of arthritis can elucidate pathophysiological mechanisms and identify novel drugs or treatments. These models allow for the examination of specific aspects of rheumatoid arthritis (RA) and the assessment of drug effects in cohorts of animals exhibiting consistent severity of multi-joint arthritis, free from the influence of other medications. However, there are relatively few studies that investigate cardiac injury in arthritis animal models. Drawing on previous reports from both domestic and international literature, as well as preliminary findings from our research team, this project employs collagen-induced arthritis (CIA) rats as the research subjects. We utilize techniques such as ultrasound imaging, pathology, and molecular biology to monitor changes in inflammatory markers, cardiac function, and blood lipid levels at various stages in untreated CIA rats. The objective is to delineate the progression characteristics of early cardiovascular disease (CVD) in CIA rats, thereby providing an experimental foundation for fundamental research on RA-related cardiac dysfunction and for the early diagnosis and prevention of RA-associated cardiac disease.

## 2 Experimental materials and methods

### 2.1 Experimental materials

#### 2.1.1 Experimental animals

Healthy male SD rats, 6 weeks old, SPF-grade, totaling 120 and weighing 180 ± 20 g, were sourced from Beijing Huafu Kang Biotechnology Co., Ltd. under license number SCXK (Jing) 2019-0008. The rats were accommodated at the SPF-level experimental animal center of Guang’anmen Hospital, China Academy of Chinese Medical Sciences, where they were kept under constant conditions of 24°C, with a 12-hour light/dark cycle, and provided ad libitum access to food and water. Approval for this study was obtained from the Ethics Committee of the Medical Experimental Center of Guang’anmen Hospital, China Academy of Chinese Medical Sciences (Approval No. IACUC-GAMH-2019-001). This study used male rats to rule out the potential cyclical effects of the estrous cycle in female animals on immune responses, inflammation levels, and cardiovascular function, a common practice in preliminary research aimed at elucidating the underlying mechanisms of disease. For a detailed list of all reagents, chemicals, and laboratory equipment used in the experiment, please refer to **Tables 1** and **2**.

**Table 1:** Laboratory reagents and chemicals.

Experimental drugs and reagents	Manufacturer	Batch number/Item number
Type II Collagen from Bovine	Chondrex Inc.	20022
Complete Freund’s Adjuvant	Chondrex Inc.	7002
Neutral Formalin Fixative	Beijing Solarbio Science & Technology Co., Ltd.	G2161
Masson’s Trichrome Staining Solution	Beso	BA-4079A
Oil Red O Staining Solution	Beso	BA4081
GVA (Water-Soluble Mounting Medium)	Zhongshan Jinqiao	ZLI-9551
Ethanol	Beijing Pufei Biotechnology Co., Ltd.	PR1357
CTNT Elisa kit	Elabscience	E-EL-R0151c
OX-LDL Elisa kit	Ruixin	RX302151R
NT-proBNP Elisa kit	Elabscience	E-EL-R3023
BNP Elisa kit	Elabscience	E-EL-R0126c

**Table 2:** Laboratory equipment.

Instruments and devices	Manufacturer	Model number
Clean Bench	Sujie Purification Company	SJ-CJ-ICU
Portable Homogenizer	Germany IKA	T10BasicUltra-turrax
Electronic Scale	Zhejiang Kaifeng Company	H2
Pipette	Eppendorf	Research plus
High-Speed Centrifuge	Beckman	5430
Ultra-Low Temperature Freezer	ThermoFisher	905
Fully Automated Biochemical Analyzer	Guangzhou Dongtang Electronic Technology Co., Ltd.	DP-180
Toe Swelling Volume Measuring Device	Anhui Zhenghua Biological Instrument Equipment Co., Ltd.	KW-7C
Electronic Scale	Zhejiang Kaifeng Company	H2
Paraffin Embedding Machine	Themo Scientific	KD-P
Microtome	Themo Scientific	RM2235
Multi-Function Biochemical Analyzer	Shandong Boyue Scientific Instruments Co., Ltd.	BK-1200
Fully Automated Multi-Function Microplate Reader	Thermo Scientific	MULTISKAN MK3
Electric Constant Temperature Incubator	Tianjin Test	DH4000A

#### 2.1.2 Experimental reagents and drugs

#### 2.1.3 Laboratory equipment

### 2.2 Methods

#### 2.2.1 Establishment of the rat CIA model

The entire immunogen preparation process must be conducted within a laminar flow hood. Initially, bovine type II collagen is dissolved in 0.01 mol/L acetic acid, followed by filtration and sterilization. Subsequently, it is combined with complete Freund’s adjuvant in a 1:1 ratio and thoroughly emulsified into a water-in-oil state using a homogenizer. When introduced into water, it does not disperse, resulting in an antigen concentration of 1 mg/mL. The rats were acclimated to the rearing environment for 7 days prior to modeling. All rats, except those in the normal group, were subjected to modeling. A subcutaneous injection of 0.15 mL of type II collagen emulsion was administered at a distance of 1 cm from the tail tip on one side of the tail root of each rat. Seven days after the initial injection, an additional 0.15 mL of type II collagen emulsion was injected subcutaneously, 2 cm away from the tail tip on the dorsal side of the tail root on the opposite side, to enhance immunity. The model was successfully established 14 days later (7, 8). The normal group received the same volume of 0.9% sodium chloride solution.

#### 2.2.2 Animal grouping

All SD rats, except for the control group, underwent modeling. By approximately day 14, swelling began in the contralateral paw of rats that did not receive collagen emulsion injections. Rats that failed in modeling were excluded, and only those that were successfully modeled with CIA were selected for subsequent experiments. The successfully modeled rats were categorized into groups based on body weight using a block randomization approach, resulting in a normal group and model groups 1 through 8, each consisting of 8 rats. Subsequently, the rats’ body weight was reassessed post-grouping to confirm uniformity across all groups.

#### 2.2.3 Material collection and tissue processing

The rats were processed in batches, and their parameters were monitored following overnight fasting without water deprivation. Subsequent to pentobarbital-induced anesthesia, blood samples were obtained via a 10ml syringe from the abdominal aorta at the initial immunization (Day 0) and on Days 14, 28, 42, 56, 70, 84, 98, and 112. The blood samples were allowed to clot for 2 hours before being centrifuged at 3000 rpm for 15 minutes to isolate serum and plasma, which were then preserved at -80 °C for future analysis. The rat aortas were dissected under sterile conditions, fixed in paraformaldehyde, and stained with Oil Red O. The hearts were also collected and fixed in paraformaldehyde for pathology. Furthermore, the knee joints of one side of the rat’s hind limbs were fixed in paraformaldehyde for pathological examination.

### 2.3 Observation indicators

#### 2.3.1 Observation of joint pathology HE-stained sections

Rat hindlimbs were dissected, and the periosteal soft tissues were removed. Specimens were fixed in 4% paraformaldehyde for 48 hours, followed by a decalcification period of 6 weeks. Upon complete decalcification, the specimens were embedded in paraffin and sectioned. Hematoxylin-eosin staining (HE staining) was conducted as follows: Bone tissue was routinely embedded in paraffin and sectioned into 4 μm-thick slices. All sections were then placed in xylene (I), xylene (II), and a series of ethanol solutions (100%, 95%, 80%, 75%) for dewaxing, followed by a 2-minute rinse with distilled water. The sections were subsequently immersed in hematoxylin stain for 5 minutes and rinsed with running water. Following this, the sections were treated with hydrochloric acid-alcohol for 30 seconds, then rinsed 3–5 times with distilled water. The specimens were placed in eosin for 2 minutes and dehydrated using 95% ethanol (I, II), 100% ethanol (I, II), and xylene-carbolic acid for 1 minute each. Finally, the sections were clarified with xylene (I, II), mounted with neutral resin, and examined under a microscope for photography.

#### 2.3.2 Cardiac ultrasound imaging examination

At each stage of the disease course, rats were removed from the animal housing facility. They were fasted overnight but allowed free access to water the day before the examination. The hair on the rats’ chests was removed using depilatory cream. After anesthesia with pentobarbital via intraperitoneal injection, the rats were placed in a supine position, and their limbs—which had been pre-coated with conductive gel—were secured to the examination table for ultrasound examination. The ultrasound probe was angled at 10°–30° to the sternal midline on the left side of the sternum to visualize the left ventricular long-axis view, which could be rotated clockwise by 90° to display the left ventricular short-axis view. M-mode tracings were then obtained and measured under the guidance of two-dimensional imaging. Shifting the probe slightly to the left after showing the parasternal long-axis view of the left ventricle revealed the pulmonary artery long-axis view. Doppler flow measurements were conducted at the mitral valve, aortic valve, and pulmonary valve orifices in both the parasternal long-axis view and pulmonary artery long-axis view. High-quality images were captured for subsequent analysis.

Under the section titled “Measure,” evaluate the parameters of diastolic function, including mitral valve E velocity (MVE), mitral valve A velocity (MVA), the mitral valve E/A ratio (MVE/A), ejection fraction (EF), and fractional shortening (FS). For each rat, compute the average from three separate curves and conduct a statistical analysis.

#### 2.3.3 Heart-to-body weight ratio in CIA rats

At each time point following the completion of echocardiography in CIA rats, the animals were euthanized, and the hearts were removed intact. The aortic root connection segment was preserved, while excess connective tissue on the heart surface and residual pericardial tissue at the aortic-cardiac junction were meticulously dissected away. The residual ends of the great vessels and both atrial appendages were excised. After gently compressing the ventricles to expel residual blood from the cardiac chambers, any remaining moisture on the heart surface was absorbed using sterile filter paper. Once all heart specimens were processed according to standardized protocols, the wet weight of each heart was measured using an electronic analytical balance with a precision of 0.1 mg. Simultaneously, the fasting body weight of the rats prior to sacrifice was recorded, and the heart weight-to-body weight ratio (HW/BW, units: mg/g) was calculated to assess differences in HW/BW among various groups and at different time points.

#### 2.3.4 Masson’s trichrome staining for cardiac tissue

①After dewaxing, rinse the sections in distilled water. ②Stain the nuclei with Weigert’s iron-hematoxylin solution for 5 to 8 minutes, followed by rinsing under running water for several minutes. ③ Differentiate using 1% hydrochloric acid in ethanol for several seconds, then rinse under running water for several minutes. ④Stain with acid fuchsin solution for 3 to 4 minutes, and then rinse briefly under running water. ⑤ Differentiate with 1% phosphomolybdic acid solution for approximately 5 minutes, centrifuge to remove excess solution, and directly counterstain with aniline blue solution for 5 minutes without rinsing. ⑥Rinse the sections with 1% glacial acetic acid for 1 minute. ⑦After a brief rinse, dehydrate with 95% ethanol, followed by absolute ethanol; clear with xylene, air-dry, and mount with neutral resin for microscopic examination.

#### 2.3.5 Measurement of rat lipid parameters

Rats were anesthetized with sodium pentobarbital, and blood was collected from the abdominal artery. All blood samples underwent centrifugation 30 minutes after collection. The supernatant serum was utilized to measure four lipid parameters—total cholesterol (TC), triglycerides (TG), high-density lipoprotein cholesterol (HDL-C), and low-density lipoprotein cholesterol (LDL-C)—using a fully automated biochemical analyzer.

#### 2.3.6 ELISA assay

Prepare all necessary reagents and working-concentration standards by sequentially adding 300 μl of 1× wash buffer to the microplate wells, allowing it to incubate for 30 seconds. Subsequently, introduce 100 μl of the sample into the serum sample wells, 100 μl of 2× diluted standard into the standard wells, and 100 μl of standard dilution buffer into the blank wells. Then, add 50 μl of diluted detection antibody (1:100 dilution) to each well. Proceed by covering the plate with a sealing film, gently shaking it, and incubating at room temperature for 2 hours. Following the incubation period, wash the plate six times with washing buffer. Next, add diluted horseradish peroxidase-labeled streptavidin to each well, seal with a new sealing membrane, and incubate at room temperature for 45 minutes, repeating the incubation step. Subsequently, introduce the color development substrate TMB to each well and incubate in darkness for 30 minutes before adding the stop solution.

#### 2.3.7 Oil Red O staining of rat aorta and coronary arteries

Oil Red O is a lipid-soluble dye that selectively stains neutral fats, such as triglycerides, within tissues. To prepare a stock solution, dissolve 0.25 g of Oil Red O in 50 mL of isopropyl alcohol, mix thoroughly, and allow the solution to stand at room temperature for 10 minutes. Staining Procedure: Begin by carefully dissecting the aortic adventitia and any adherent tissues, followed by fixation in a 4% paraformaldehyde solution for 2 to 3 days. After fixation, rinse extensively with distilled water for 4 hours, then immerse the tissues in 60% isopropanol for 10 minutes. Dilute the Oil Red O stock solution with distilled water at a 3:2 volume ratio, allow it to stand for 10 minutes, and then place the arteries in the stain for 3 to 4 hours. Following staining, differentiate the tissues in 60% isopropanol 5 to 6 times until the aortic region appears white and transparent, with distinct red staining in the plaque areas. Rinse the samples three times with distilled water, examine the aorta under a microscope, and then fix and photograph the results.

#### 2.3.8 Statistical analysis

Data analysis was conducted using SPSS version 22.0, with results presented as mean ± standard deviation (± s). Initially, normality and homogeneity of variance were assessed. When data satisfied normality assumptions, independent samples t-tests were employed to compare differences between two groups. For comparisons of means across multiple groups, one-way analysis of variance (ANOVA) was utilized. Normality was evaluated using the Shapiro-Wilk test, while homogeneity of variance was determined through Levene’s test. *Post-hoc* comparisons following ANOVA utilized LSD when variances were homogeneous and Dunnett’s T3 when variances were heterogeneous. Non-normally distributed data were analyzed using the Mann-Whitney U test for two groups or the Kruskal-Wallis test for multiple groups. Graphs were created using GraphPad Prism software. A p-value of less than 0.05 was deemed statistically significant (*P < 0.05, **P < 0.01).

## 3 Experimental results

### 3.1 Changes in joint pathology at different stages of CIA rat disease progression

**Figure 1:**
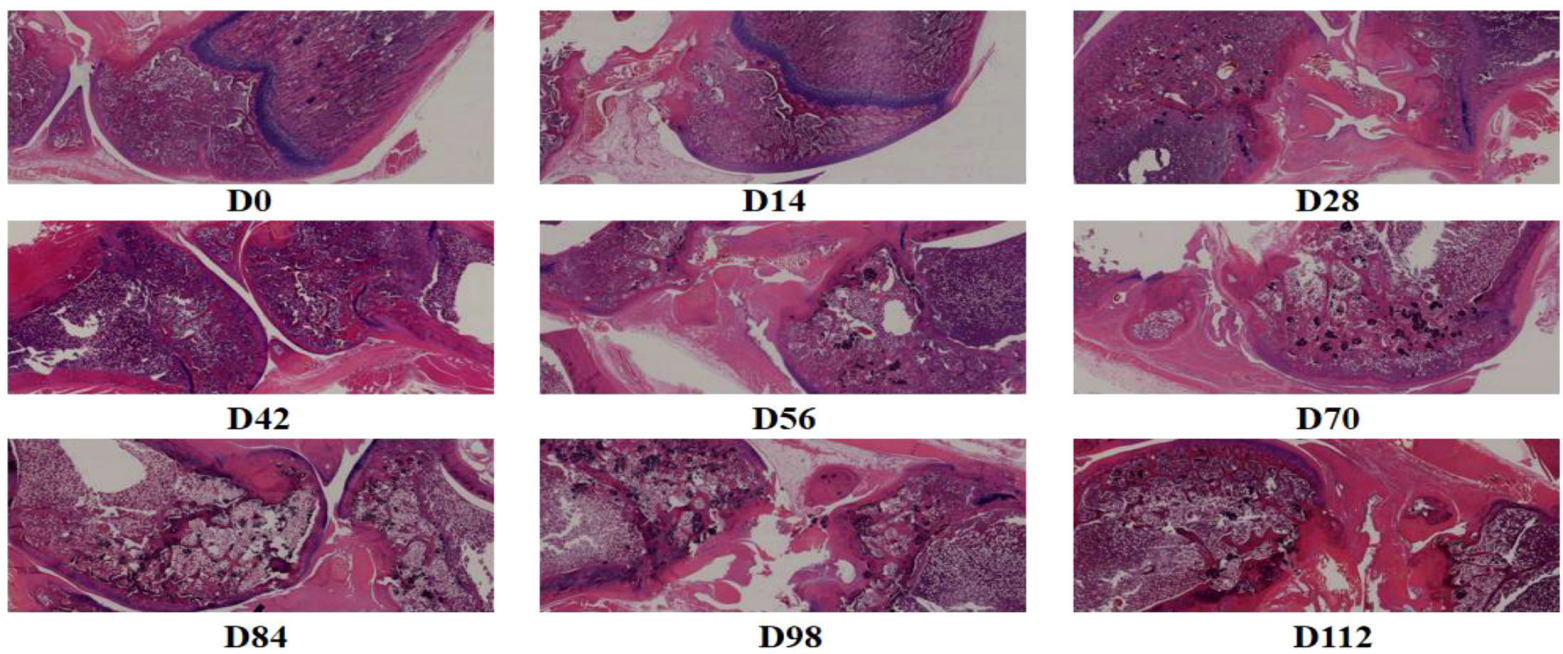
Pathological changes in joints of CIA rats at different disease stages.

Semi-quantitative pathological scoring of HE-stained ankle joint sections from rats at various time points indicated that the joints of rats in the normal control group were structurally intact, exhibiting no inflammatory cell infiltration, vascularization, or bone destruction. Throughout the observation period, joint inflammation scores remained low (mean score < 0.5) (**Table 3**). In the CIA model group, notable synovial hyperplasia and minimal inflammatory cell infiltration were observed as early as 14 days post-immunization (D14), leading to a significant increase in the joint inflammation score. Inflammation peaked between D28 and D42, characterized by extensive inflammatory cell infiltration, pronounced vascularization, and localized joint bone erosion, with scores of 7.92 ± 0.81 and 8.25 ± 0.76, respectively, both significantly higher than those in the control group (P < 0.01). Following D56, the CIA model transitioned into the chronic inflammatory phase, during which the degree of inflammation decreased slightly but remained elevated. Vascularization and progressive bone erosion continued; by the end of the observation period at D112, inflammation had not fully resolved, reflecting the chronic, recurrent inflammatory nature of collagen-induced arthritis (**Figure 1**).

**Table 3:** Semi-quantitative pathological scores of joint inflammation in CIA rats at different time points (x ± s, n = 8 per group per time point).

Time point	Control group score (points)	CIA model group score (points)
D0	0.22 ± 0.15	0.28 ± 0.18
D14	0.31 ± 0.12	2.76 ± 0.53*
D28	0.35 ± 0.16	7.92 ± 0.81**
D42	0.41 ± 0.19	8.25 ± 0.76**
D56	0.38 ± 0.21	6.17 ± 0.68**
D70	0.42 ± 0.17	5.83 ± 0.72**
D84	0.39 ± 0.20	5.32 ± 0.65**
D98	0.44 ± 0.18	5.14 ± 0.62**
D112	0.40 ± 0.17	4.98 ± 0.59**

A semi-quantitative scoring system ranging from 0 to 10 was used to evaluate joint pathology based on four criteria: inflammatory cell infiltration, synovial hyperplasia, vascularization, and bone erosion. A higher total score indicates more severe inflammatory damage. * indicates P < 0.05 compared with the control group at the same time point; ** indicates P < 0.01 compared with the control group at the same time point.

### 3.2 Cardiac ultrasound imaging examination

#### 3.2.1 Changes in cardiac diastolic function at different disease stages in CIA rats

**Figure 2:**
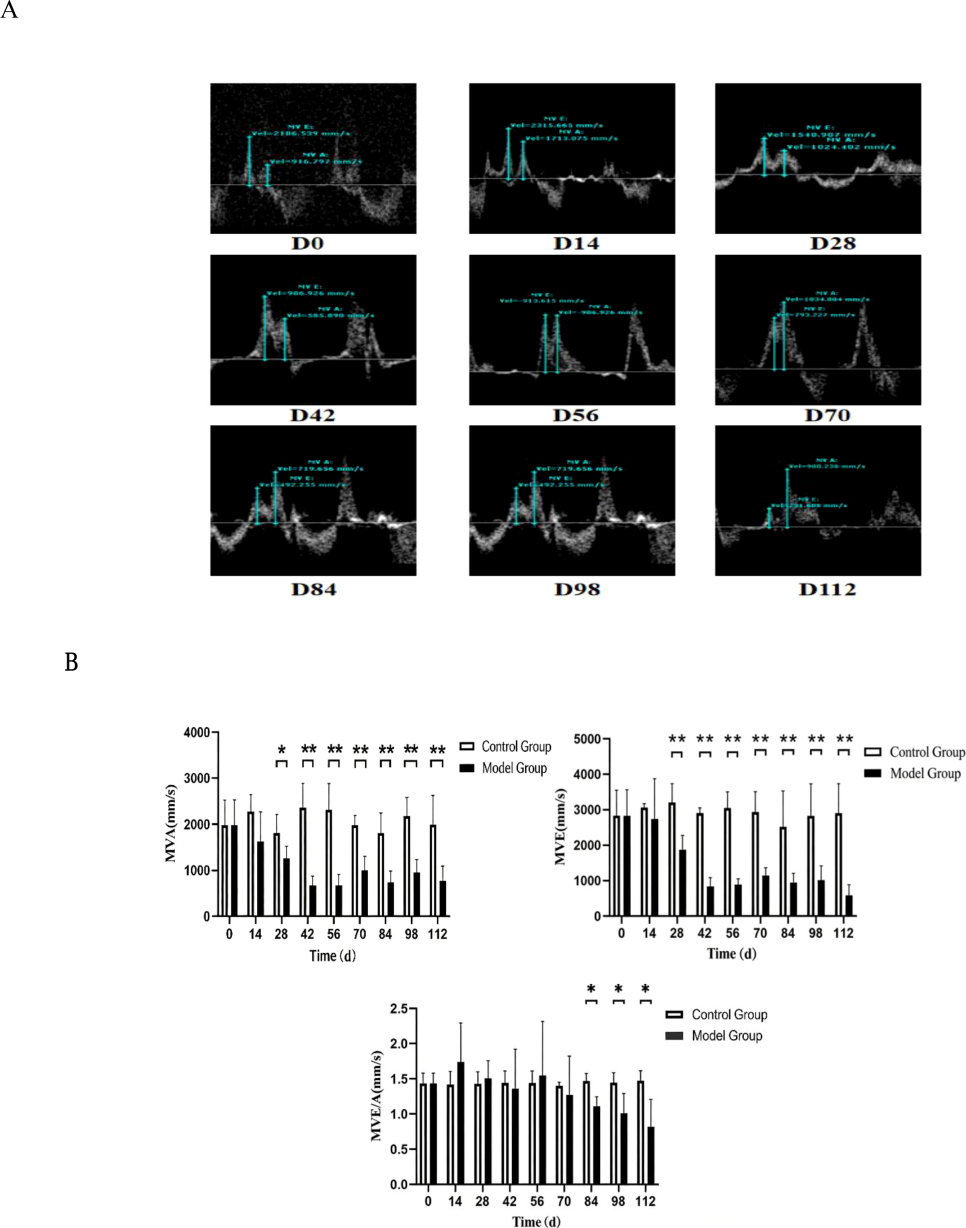
Echocardiographic alterations in CIA rats at various disease stages. **(A)** Assessment of cardiac diastolic function via echocardiography at different disease stages in CIA rats; **(B)** Comparative analysis of diastolic function among the various groups. Compared with the normal group, *P < 0.05, **P < 0.01.

After administering anesthesia to rats at various disease stages, cardiac function was dynamically monitored using a small animal ultrasound imaging system. MVA denotes the A peak of the mitral valve orifice blood flow velocity, which exhibited a gradual decline over time. Compared to the normal group, values from D28 to D112 were significantly lower (P < 0.05). MVE represents the E peak of the mitral valve orifice blood flow velocity, which also decreased progressively over time, with D28 to D112 showing significantly lower values than the normal group (P < 0.01). In normal rats, MVE was greater than 1, while the mean distribution of MVE/A in D56 CIA rats was approximately 1. The MVE/A ratio began to fall below 1 in D70 CIA rats. Nevertheless, the overall mean remained above 1. In comparison to the normal group, the MVE/A ratio at D84 was significantly lower (P < 0.05) (**Figures 2A, B**). According to the statistical analysis, abnormalities in diastolic cardiac function could be detected by ultrasound imaging starting from D84 in the CIA rat model (MVE/A < 1).

#### 3.2.2 Changes in cardiac contractility at different stages of CIA rat disease progression

**Figure 3:**
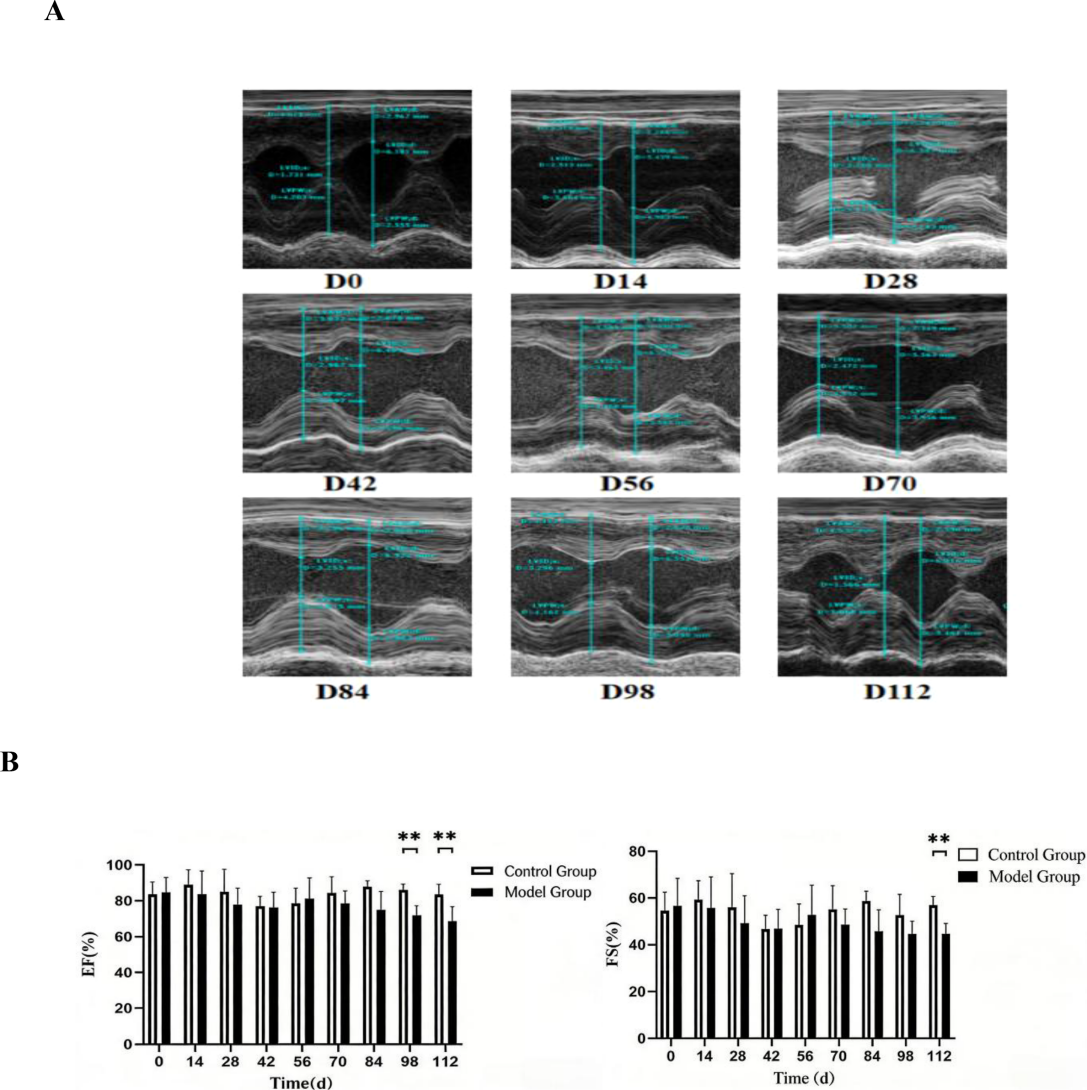
Echocardiographic alterations in CIA rats at various disease stages. **(A)** Assessment of cardiac systolic function via echocardiography at distinct disease stages in CIA rats; **(B)** Comparative analysis of systolic function among different groups. Compared with the normal group, **P < 0.01. Compared with age-matched normal rats, no significant differences in ejection fraction (EF) or fractional shortening (FS) were observed in the initial statistical analysis. However, EF decreased significantly compared to the control group between days 98 and 112 (P < 0.01), and FS decreased significantly compared to the control group on day 112 (P < 0.01).

Following the anesthetization of rats at various modeling stages, cardiac contraction function was evaluated by recording cardiac cycles using M-mode. Three cardiac cycles per rat were averaged and subsequently analyzed with the Measure function. The analysis indicated a prolonged duration of cardiac contraction abnormalities. In comparison to age-matched normal rats, no significant differences in ejection fraction (EF) or fractional shortening (FS) were noted during early-stage statistical analysis. However, EF exhibited a significant decline relative to the normal group between days 98 and 112 (P < 0.01), and FS demonstrated a significant decrease compared to the normal group at day 112 (P < 0.01) (**Figures 3A, B**). These findings suggest that ultrasound imaging is capable of detecting the onset of systolic cardiac dysfunction.

### 3.3 Changes in the heart-to-body weight ratio at different stages of the disease course in CIA rats

**Figure 4:**
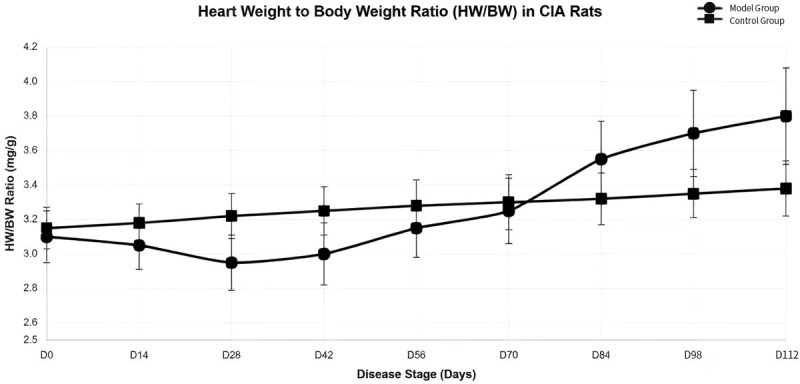
Changes in the heart-to-body weight ratio in CIA Rats at different stages of the disease.

CIA rats were euthanized at various stages of the disease progression, and the heart weight-to-body weight ratio (HW/BW) was calculated. In the normal group, the HW/BW ratio exhibited a gradual increase. In contrast, the HW/BW ratio in the model group decreased progressively from D0 to D28. From D28 to D56, the HW/BW ratio began to rise gradually; by approximately D56–D70, the HW/BW ratio in CIA rats fluctuated at levels comparable to those of normal rats. From D84 to D112, the HW/BW ratio in CIA rats was significantly elevated compared to the normal group (**Figure 4**), indicating the onset of myocardial hypertrophy in CIA rats.

### 3.4 Changes in myocardial fibrosis at different stages of CIA rat disease progression

**Figure 5:**
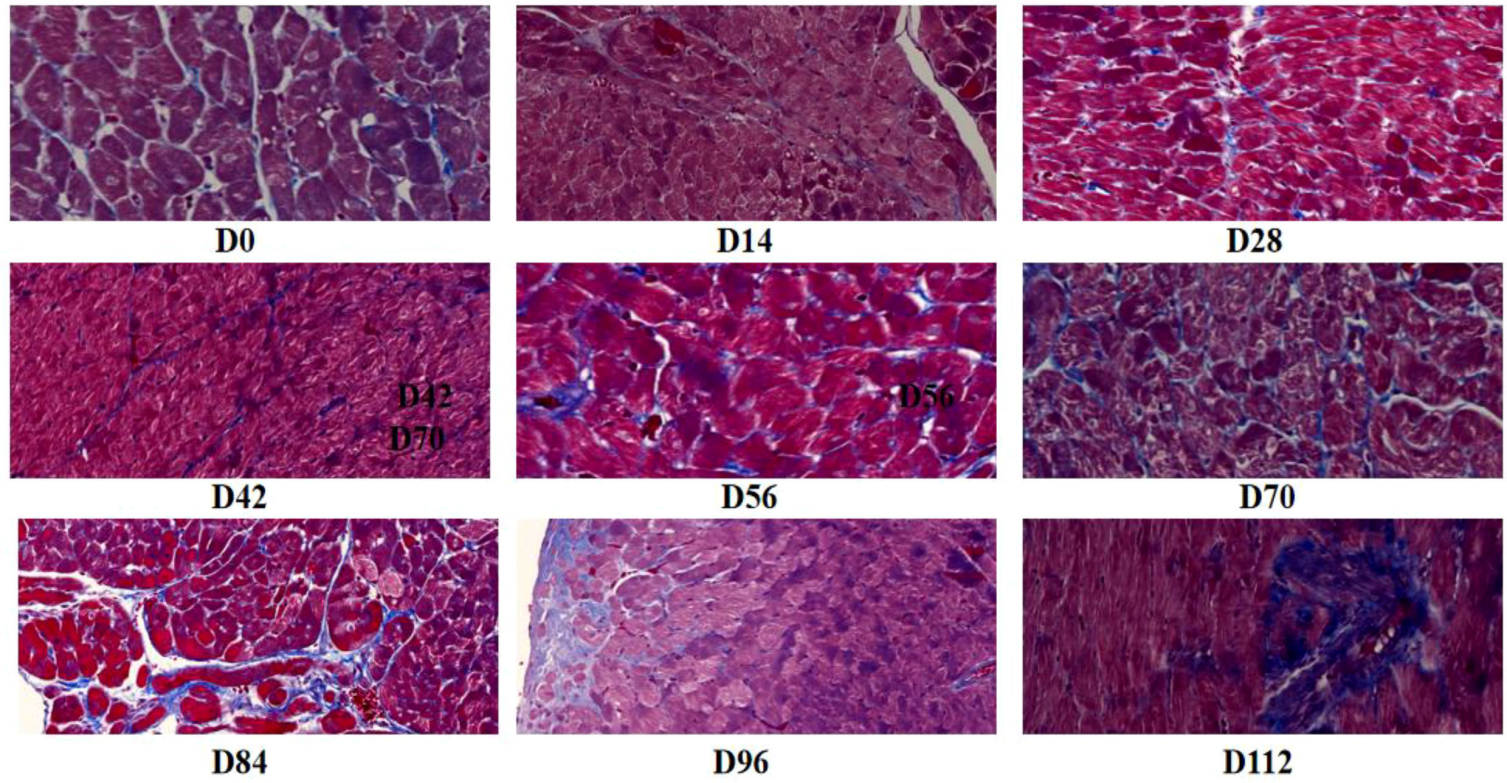
Representative Masson’s trichrome staining of hearts from CIA rats at different disease stages.

Paraffin sections of myocardial tissue were stained with Masson’s trichrome stain, resulting in myocardial cells appearing purple-red and collagen staining blue. As illustrated in the figure, CIA rats demonstrated significant collagen deposition in myocardial tissue beginning on day 56 post-modeling, in contrast to the normal group (**Figure 5**). With disease progression, the extent of cardiac fibrosis continued to deteriorate.

### 3.5 Lipid profile analysis in CIA rats

**Figure 6:**
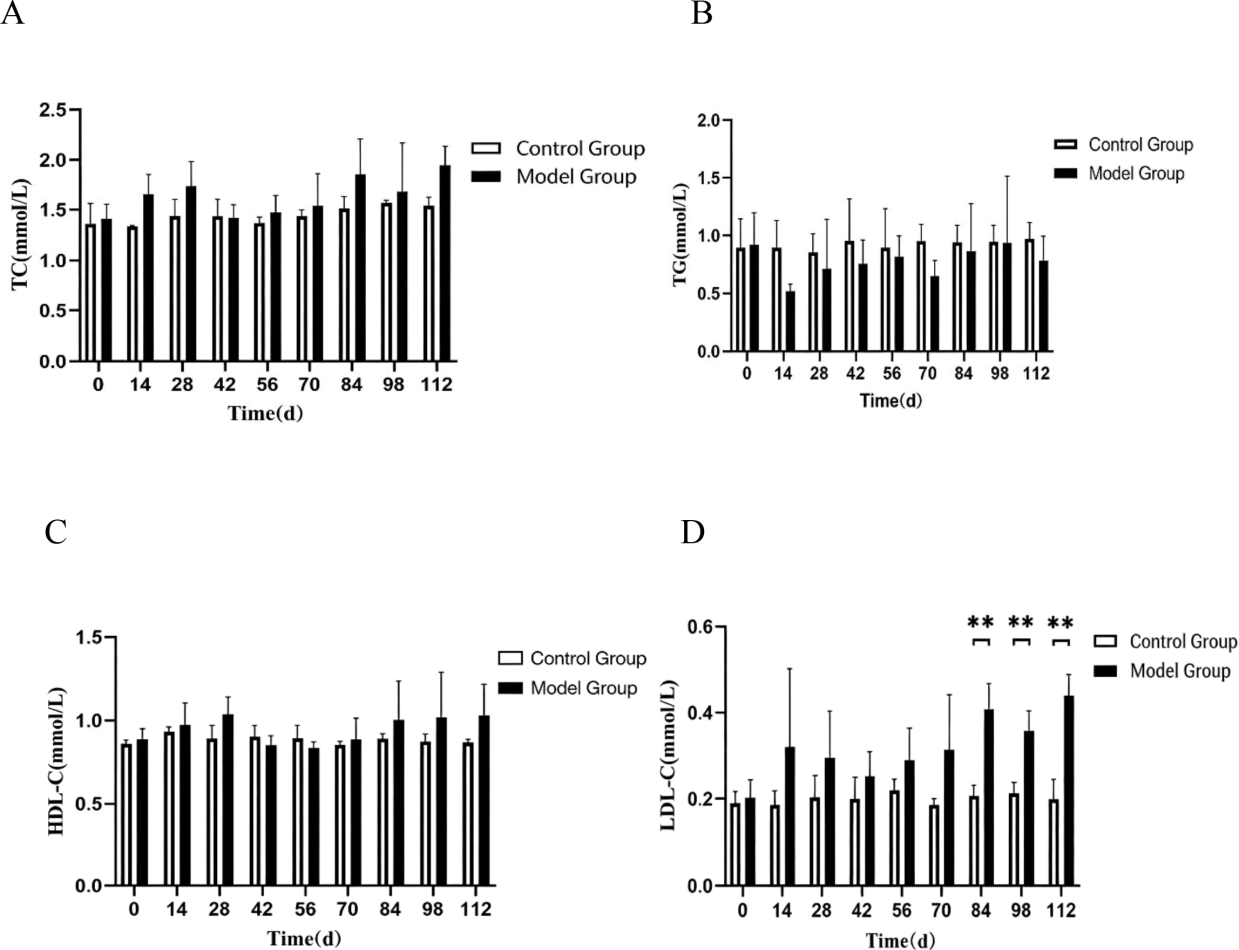
Illustrates alterations in lipid profiles in CIA rats across various stages of the disease. **(A)** Total cholesterol (TC) levels in CIA rats at distinct disease stages; **(B)** Triglyceride (TG) levels in CIA rats at different disease stages; **(C)** High-density lipoprotein cholesterol (HDL-C) levels in CIA rats at various disease stages; **(D)** Low-density lipoprotein cholesterol (LDL-C) levels in CIA rats at different disease stages. Note: Compared with the normal group, **P < 0.01. However, BNP levels at D112 were significantly elevated compared to the normal group (P < 0.01), indicating a potential risk of heart failure. Compared to the normal group, NT-proBNP levels did not show a statistically significant difference. Additionally, OX-LDL levels rose significantly from D98 to D112 compared to the normal group (P < 0.05).

CIA rats were euthanized at various time points throughout the disease progression, and blood samples were collected for subsequent analysis. The model group demonstrated elevated total cholesterol (TC) levels compared to the normal group during the D14-D28 and D56-D112 intervals; however, these differences were not statistically significant (**Figure 6A**). In contrast, triglyceride (TG) levels in the model group remained consistently lower than those in the normal group, with no significant differences observed (**Figure 6B**). High-density lipoprotein cholesterol (HDL-C) levels in the model group also showed no significant variation when compared to the normal group (**Figure 6C**). Notably, low-density lipoprotein cholesterol (LDL-C) levels significantly increased from D84 to D112 (P < 0.01), suggesting an elevation in cardiovascular disease (CVD) risk factors (**Figure 6D**).

### 3.6 ELISA detection of serum CTNT, BNP, NT-proBNP, and OX-LDL levels

**Figure 7:**
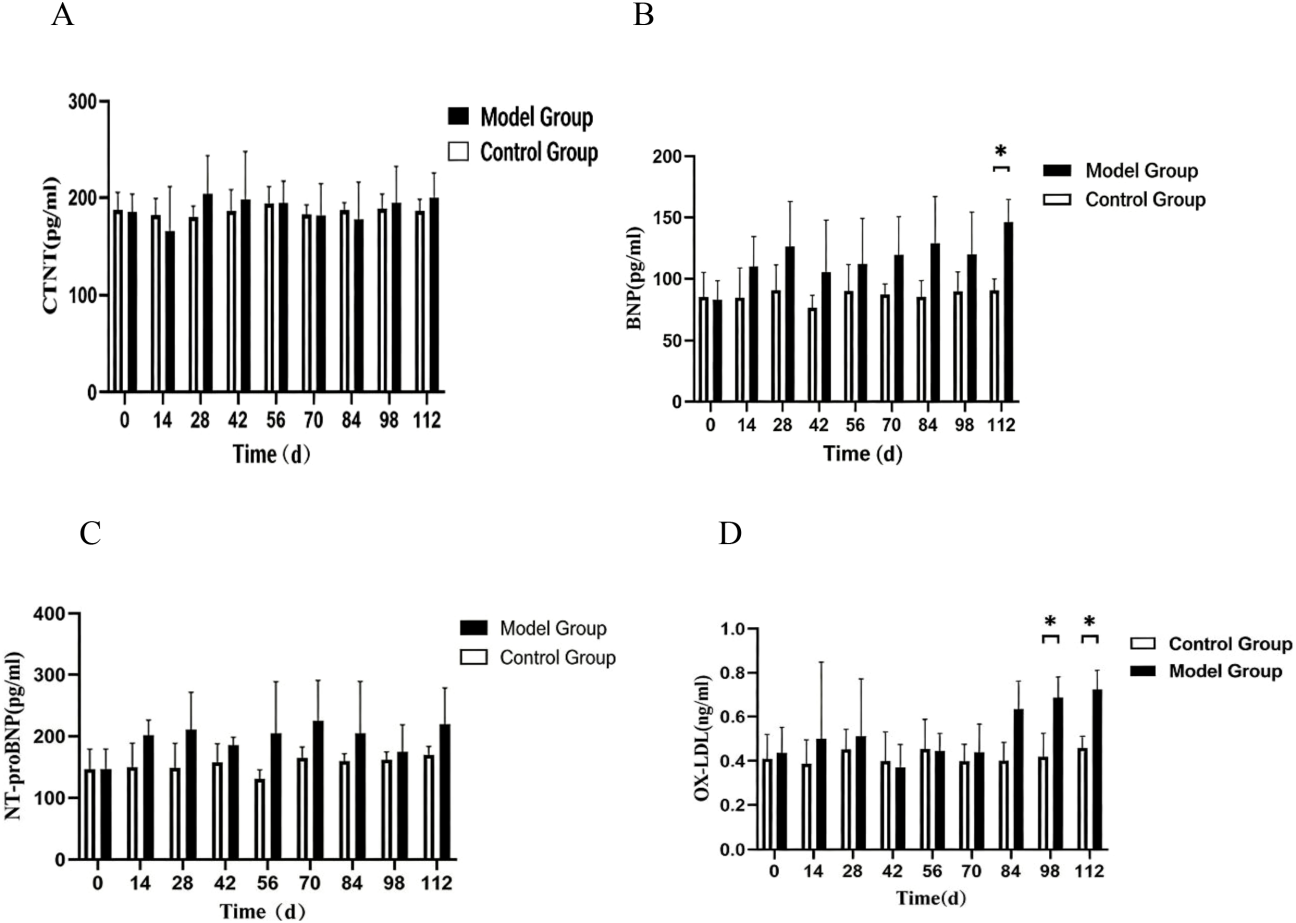
illustrates the alterations in cardiac function indicators in CIA rats across various disease stages. **(A)** Depicts the variations in CTNT levels in CIA rats at different disease stages; **(B)** shows the changes in BNP levels in CIA rats at different disease stages; **(C)** presents the fluctuations in NT-proBNP levels in CIA rats at different disease stages; **(D)** highlights the modifications in OX-LDL levels in CIA rats at different disease stages. Note: Compared with the control group, *P < 0.05, **P < 0.01.

CIA rats were euthanized at various stages of disease progression, and blood samples were collected for analysis. Compared with the normal group, CTNT levels did not show a statistically significant difference (**Figure 7A**). However, BNP levels were significantly elevated at D112 relative to the normal group (P < 0.01) (**Figure 7B**), indicating a potential risk of heart failure. NT-proBNP levels did not show a significant statistical difference compared to the normal group (**Figure 7C**). Furthermore, OX-LDL levels were significantly increased from D98 to D112 when compared to the normal group (P < 0.05), suggesting a cardiovascular disease risk in CIA rats.

### 3.7 Changes in Oil Red O staining of the thoracic aorta in CIA rats at different disease stages

**Figure 8:**
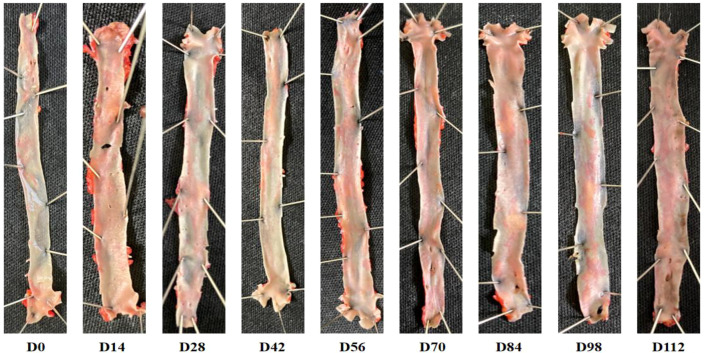
Aortas stained with Oil Red O at different stages of CIA rat disease progression.

**Figure 9:**
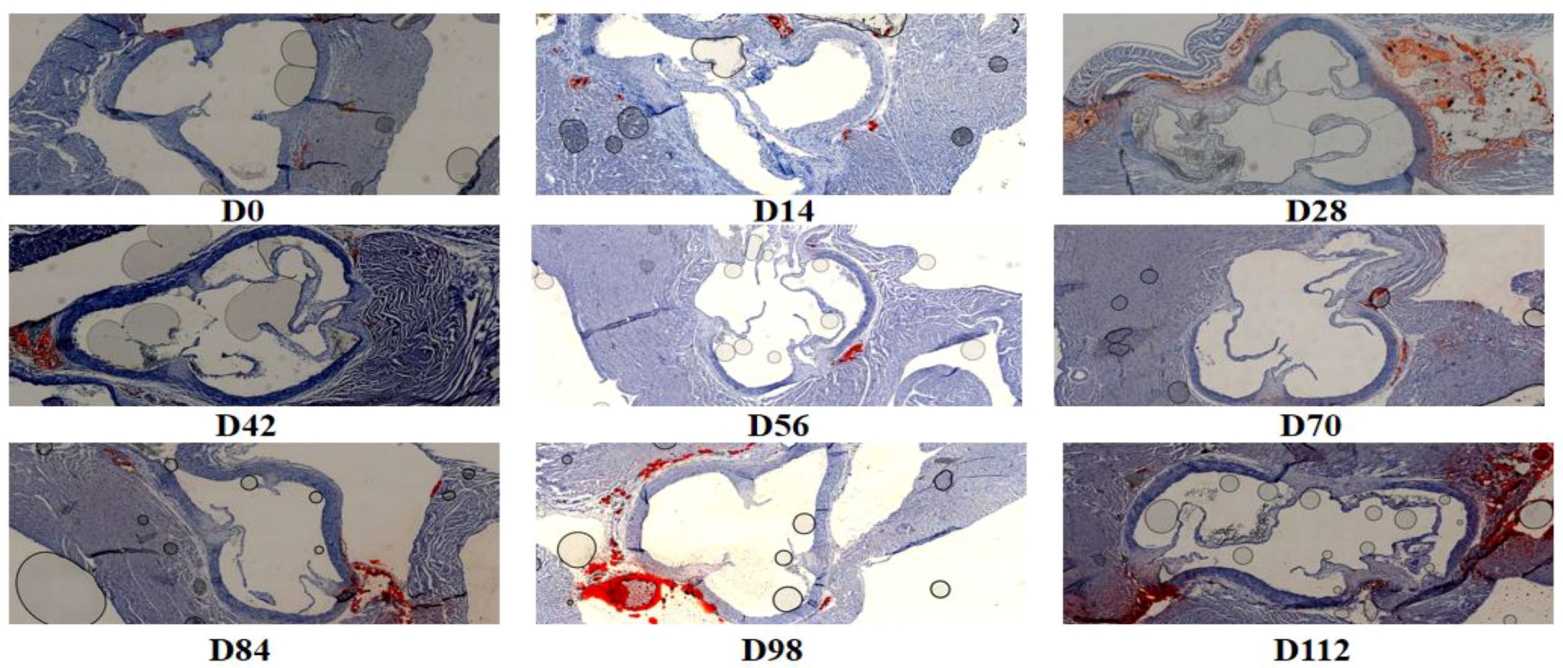
Oil Red O staining of coronary arteries in CIA rats at different stages of disease progression.

Aortas were isolated from normal rats and rats with collagen-induced arthritis (CIA) at various disease stages. Oil Red O staining was conducted on the arterial intima. The results revealed that CIA rats’ aortas showed no red staining at any disease stage up to D112 (**Figure 8**), suggesting the absence of atherosclerotic plaque formation. Similarly, no red staining was detected in the coronary arteries of CIA rats at any disease stage up to D112 post Oil Red O staining, (**Figure 9**) indicating the lack of atherosclerotic plaque formation in their coronary arteries.

## 4 Discussion

As rheumatoid arthritis (RA) advances, it can impact various organs throughout the body. The heart, rich in connective tissue, is particularly susceptible to varying degrees of cardiac involvement. The most prevalent cardiac manifestation is pericarditis. Inflammation of the joints can intensify the inflammatory process associated with RA, potentially elevating the risks of myocardial infarction and heart failure, which significantly impair patients’ quality of life and survival. Compared to the general population, the risks of myocardial infarction and heart failure are 1.67% and 1.87%, respectively (9). Therefore, preventing, managing, and enhancing the prognosis of cardiovascular disease (CVD) in RA patients is of paramount importance. Current clinical strategies addressing the onset, progression, and treatment of RA alongside CVD primarily emphasize diagnostic techniques and clinical management. However, reports on animal models of RA and the progression of CVD in RA patients remain limited.

This study did not directly measure classic systemic inflammatory markers, such as circulating CRP, IL-6, and TNF-α, which constitutes a limitation. Nevertheless, the local joint inflammatory pathology scores obtained at various time points can serve as effective surrogate markers for the systemic inflammatory burden in CIA rats. Synovial inflammation is the primary site of pro-inflammatory cytokine production in both the CIA model and human RA. Furthermore, the severity and timeline of local inflammation directly reflect the overall exposure levels of circulating pro-inflammatory factors.

The temporal analysis conducted in this study indicates that joint inflammation in CIA rats peaked between D28 and D42, subsequently entering a phase characterized by persistent chronic inflammation. The cardiac structural and functional abnormalities identified exhibited a clear time-lagged association: myocardial fibrotic changes were first detectable as early as D56, while cardiac diastolic dysfunction emerged at D84. Systolic dysfunction further deteriorated after D98, and the standardized heart weight-to-body weight ratio was significantly elevated compared to the control group starting from D84. This observed temporal relationship suggests that persistent local joint inflammation drives a state of chronic systemic inflammation, which progressively induces structural remodeling and functional impairment in the heart, a distant organ. These findings support the hypothesis that “inflammatory spillover” is a key mechanism underlying the pathogenesis of cardiovascular complications in chronic arthritis.

Numerous studies have established that pro-inflammatory cytokines resulting from synovial inflammation in rheumatoid arthritis (RA) and collagen-induced arthritis (CIA) models can enter the bloodstream and contribute to cardiac damage via various direct and indirect mechanisms. Among these cytokines, TNF-α can directly induce cardiomyocyte hypertrophy, promote the proliferation and activation of cardiac fibroblasts, accelerate collagen deposition and myocardial fibrosis, and impair coronary endothelial function, leading to microcirculatory disorders that further exacerbate cardiac ischemic damage (10). Similarly, IL-6 can stimulate hepatocytes to synthesize acute-phase reactants such as C-reactive protein (CRP), while also activating the renin-angiotensin system, which promotes vasoconstriction and myocardial remodeling, thereby driving the progression of myocardial hypertrophy and fibrosis (11). Clinical epidemiological studies have confirmed that persistent high disease activity, indicative of a sustained inflammatory burden, in RA patients is significantly correlated with an increased risk of cardiovascular events. Achieving target levels of inflammation through optimal treatment can substantially reduce the risk of cardiovascular complications in individuals with RA (12).

This study establishes a spatiotemporal association between local joint inflammatory activity and cardiac phenotypes, providing indirect yet compelling support for the mechanism by which chronic inflammatory burden induces cardiac injury. It significantly clarifies the relationship between cardiac injury and inflammatory status in CIA rats. Future research will incorporate the detection of circulating inflammatory cytokines to elucidate the specific regulatory roles of various pro-inflammatory factors in cardiac injury among CIA rats.

The pathophysiology of ischemic disease exhibits notable differences between patients with rheumatoid arthritis (RA) and those without. RA patients are more prone to experience silent or unrecognized ischemia, indicating that subclinical alterations in the coronary microcirculation may be mechanistically associated with atherosclerosis (AS) in larger vessels (13). Acute systemic inflammation can provoke cardiac inflammation, which may subsequently lead to myocardial fibrosis (14), as well as contractile and diastolic dysfunction, and arrhythmias (15). Ultrasound imaging serves as a relatively noninvasive and safe diagnostic modality, offering substantial value for monitoring disease progression and facilitating early detection of cardiac functional and structural changes. Cardiac ultrasound imaging in collagen-induced arthritis (CIA) rats revealed significant findings regarding diastolic function: the mitral valve E-wave velocity (MVE) progressively decreased over time, with a notable reduction observed at days 28 to 112 compared to the control group (P < 0.01). In normal rats, MVE exceeds mitral valve A-wave velocity (MVA). By day 56 in CIA rats, the mean MVE/A ratio was approximately 1.By day 70 (D70), CIA rats exhibited MVE < MVA and MVE/A < 1, although the overall mean remained greater than 1. By day 84 (D84), the MVE/A ratio was significantly reduced compared to the normal group (P < 0.05) (**Figures 2A, B**). These findings indicate that diastolic cardiac dysfunction (MVE/A < 1) became detectable by ultrasound imaging in CIA rats starting at D84. It is noteworthy that while the peak velocity of the E wave (MVE), indicative of early ventricular filling, and the peak velocity of the A wave (MVA), indicative of atrial systolic filling, were both significantly lower than those observed in the control group beginning on day 28, the ratio of these two measures (MVE/A) did not exhibit a statistically significant decrease until day 84. This observation suggests that in the early stages of chronic ischemic heart disease (CIA) (D28–D70), left ventricular diastolic function had already begun to deteriorate, as evidenced by a reduction in overall diastolic filling velocity. However, the degree of impairment in early diastole (E-wave) and late diastole (A-wave) may have progressed in a relatively parallel manner, resulting in no significant alteration in the ratio. As the disease advanced beyond day 84, early diastolic function may have experienced further selective impairment, or atrial compensatory function may have been relatively enhanced, leading to a decrease in the E/A ratio and the emergence of a characteristic pattern of diastolic dysfunction. This transition from “overall reduced filling” to “abnormal E/A ratio” may reflect the dynamic process of progressive decline in myocardial compliance and a gradual increase in left ventricular filling pressure. The observation of systolic dysfunction suggested a longer disease course. In early statistical analyses of cardiac systolic function, no significant differences in ejection fraction (EF) or fractional shortening (FS) were observed when compared with age-matched normal rats. However, EF showed a significant decrease relative to the control group between D98 and D112 (P < 0.01), and FS decreased significantly at D112 (P < 0.01) (**Figures 3A, B**). These results indicate that ultrasound imaging can effectively detect the onset of systolic dysfunction. This finding aligns with the European Society of Cardiology guidelines classification (16), which states that rheumatoid arthritis (RA) patients most commonly present with preserved ejection fraction heart failure rather than reduced ejection fraction heart failure (17). Furthermore, non-ischemic heart failure is more prevalent in RA than ischemic heart failure (18).

CIA rats were euthanized at various stages of disease progression. Their hearts were excised and weighed, allowing for the calculation of the heart weight-to-body weight ratio (HW/BW). In the normal group, the HW/BW ratio exhibited a gradual increase. In contrast, the HW/BW ratio in the model group decreased progressively from D0 to D28. Subsequently, from D28 to D56, the HW/BW ratio began to rise gradually. By approximately D56–D70, the HW/BW ratio in CIA rats stabilized at levels comparable to those of normal rats. However, from D84 to D112, the HW/BW ratio in CIA rats was significantly elevated compared to the normal group (**Figure 4**), indicating the onset of myocardial hypertrophy in CIA rats. Histological examination of paraffin-embedded myocardial tissue sections stained with Masson’s trichrome revealed significant collagen deposition in the myocardium of CIA rats starting on modeling day D56, compared to the normal group (**Figure 5**). As the disease progressed, cardiac fibrosis intensified. In line with these findings, recent studies utilizing cardiac magnetic resonance imaging (MRI) and positron emission tomography-computed tomography (PET-CT) have documented cardiac inflammation and fibrosis in asymptomatic RA patients lacking cardiovascular risk factors during the early stages of the disease (19, 20). However, the relationship between cardiac injury and clinical or systemic inflammation remains contentious, and the temporal sequence of these events is not well understood. For instance, associations have been noted between non-ischemic heart failure and peripheral markers of inflammation or disease activity (21), yet no correlation has been established between the risk of RA-related myocardial infarction and disease activity (22).

Dyslipidemia is prevalent among patients with active rheumatoid arthritis (RA), who exhibit an increased risk of cardiovascular disease (CVD) at relatively low cholesterol levels compared to the general population without RA. The underlying mechanisms contributing to these paradoxical alterations in the lipid profiles of RA patients remain unclear, and the interplay among lipid fractions, inflammation, and CVD risk in RA appears to be highly complex (23). Collagen-induced arthritis (CIA) rats were euthanized at various stages of disease progression, and blood samples were collected for analysis. Compared to the normal group, the model group demonstrated higher total cholesterol (TC) levels during the D14-D28 and D56-D112 intervals, although these differences were not statistically significant (**Figure 6A**). Triglyceride (TG) levels in the model group were consistently lower than those in the normal group, also without significant differences (**Figure 6B**). High-density lipoprotein cholesterol (HDL-C) levels in the model group did not differ significantly from those in the normal group (**Figure 6C**); however, low-density lipoprotein cholesterol (LDL-C) levels were significantly elevated (P < 0.01) during the D84-D112 period compared to the normal group, indicating increased CVD risk factors (**Figure 6D**). CIA rats show increased levels of LDL-C and OX-LDL, resembling the lipid metabolism irregularities observed in human RA. Nevertheless, there was no decrease in HDL-C, indicating that interspecies distinctions could constrain the translational value of this model. An Indian study reported dyslipidemia in 38.5% of RA patients, with LDL-C being the most prevalent abnormality (24). Additionally, a Pakistani study found overall dyslipidemia in 44.87% of patients with various autoimmune diseases (25). A paradoxical lipid profile has been reported to triple the cardiovascular disease (CVD) risk in patients (26). Notably, ethnic variations in lipid profiles contribute to heightened CVD risk and elevated inflammation levels in patients with rheumatoid arthritis (RA), as the disease itself exacerbates atherosclerosis (AS) (27). Patients with RA may benefit from more stringent lipid targets than those typically recommended for conventional CVD risk prevention and intervention (28). Evidence indicates that lipid-lowering therapy possesses significant anti-inflammatory and immunomodulatory properties, which may yield beneficial effects for RA (29).

B-type natriuretic peptide (BNP) and N-terminal pro-B-type natriuretic peptide (NT-proBNP) serve as independent predictors of cardiovascular morbidity and all-cause mortality, including among patients with rheumatoid arthritis. ELISA results indicated significantly elevated BNP levels at Day 112 when compared to the control group (P<0.01), whereas NT-proBNP did not exhibit a statistically significant difference relative to the control group (**Figures 7B, C**). In cases of early inflammatory polyarthritis, NT-proBNP correlates with Health Assessment Questionnaire (HAQ) scores and C-reactive protein (CRP) levels (30), suggesting a potential association between joint and cardiac (myocardial or vascular) structural damage induced by chronic inflammatory diseases. This study revealed that in the late stages of the disease (D112), levels of BNP—an active hormone indicative of ventricular wall stress—were significantly elevated, while its inactive N-terminal fragment, NT-proBNP, did not exhibit a corresponding significant change. This discrepancy may arise from several factors. First, BNP and NT-proBNP possess different metabolic clearance rates and half-lives in the body, with BNP having a half-life of approximately 20 minutes and NT-proBNP ranging from 60 to 120 minutes. This difference may lead to a time lag between their dynamic changes at the time of measurement. Second, in early or mild heart failure, alterations in BNP secretion may be more sensitive than those in NT-proBNP. Finally, in RA-associated cardiomyopathy, the mechanisms or regulatory patterns governing BNP release may differ from those observed in typical ischemic heart failure. The significant elevation in BNP noted at the disease endpoint in this study was consistent with concurrent systolic dysfunction, as evidenced by decreased EF and FS, collectively indicating a risk of cardiac decompensation at D112.In a study investigating the evolving cardiac function in a well-established rheumatoid arthritis animal model, researchers noted compromised cardiac performance linked to prolonged elevation of blood epinephrine levels in CIA rats. This effect was primarily attributed to β1AR-mediated excitation-contraction coupling signaling. Moreover, there was a positive correlation between serum epinephrine levels and NT-proBNP, a recognized heart failure biomarker in individuals with rheumatoid arthritis (31). The advent of high-sensitivity cardiac troponin assays has improved the detection of low levels of circulating cardiac troponin, facilitating the identification of subclinical myocardial injury. C-terminal N-terminal troponin T (CTNT) has emerged as the preferred biomarker for noninvasive detection of myocardial injury and is found to be elevated in various diseases, excluding acute coronary syndromes. ELISA results from this study demonstrated no significant difference in CTNT levels when compared to the normal group. Low-density lipoprotein (LDL) oxidation results in oxidized LDL (OX-LDL), which can further provoke inflammatory responses while being influenced by the inflammatory milieu, thereby contributing to the development of atherosclerosis (AS). ELISA results revealed significantly elevated OX-LDL levels in collagen-induced arthritis (CIA) rats at Day 98 compared to the normal group (P<0.05). Potential explanations for the lipid paradox include rheumatoid arthritis-related immune dysregulation, inflammation, and oxidative stress pathways (32), which may lead to reduced lipid levels in rheumatoid arthritis patients while simultaneously increasing the risk of cardiovascular disease.

Oil Red O is a lipophilic dye that exhibits high solubility in fats. Its staining mechanism relies on the specific adsorption to neutral triglycerides, lipids, and lipoproteins present in tissues and cells, which facilitates the visualization of fats. Oil Red O staining serves as a method for detecting alterations in large-vessel arterial plaques. Results from Oil Red O staining of the D112 aortic and coronary arteries revealed no significant red plaque formation, indicating that CIA rats did not develop substantial atherosclerotic plaques during the early stages of cardiac dysfunction. Although no macroscopic atherosclerotic plaques are evident, microvascular endothelial dysfunction may play a role in early CVD (33).

In brief, CIA rats initially develop diastolic dysfunction, followed by systolic dysfunction, along with noticeable myocardial hypertrophy and fibrosis. Lipid-related markers indicated a significant increase in LDL-C levels from D84 to D112 (P<0.01), and OX-LDL levels from D98 to D112 were notably higher than those of the control group (P<0.05), suggesting a substantially elevated risk of CVD in CIA rats (**Figure 7D**). CIA rats displayed higher cardiac weights compared to the controls, indicating myocardial hypertrophy. Masson staining of CIA rat hearts revealed a progressive deterioration of myocardial fibrosis throughout various disease stages. Conversely, Oil Red O staining of the aorta and coronary arteries at D112 showed no positive findings, indicating the absence of significant atherosclerotic plaques in the early stages of cardiovascular disease in CIA rats. This research offers empirical support for an animal model in the fundamental investigation of RA complicated by CVD, providing a valuable reference for such studies.

This study has several limitations that warrant acknowledgment. A primary limitation is the exclusive use of male animals. Given that the majority of clinical rheumatoid arthritis (RA) patients are women, the generalizability of our findings to clinical practice—especially for female patients—is constrained. Estrogen is known to have modulatory effects on both the immune and cardiovascular systems. Future studies should include female collagen-induced arthritis (CIA) animals to ascertain whether the temporal patterns of cardiac lesions observed in this study are applicable across genders and to investigate potential gender-specific mechanisms. Additionally, the assessment of myocardial hypertrophy was primarily based on the heart-to-body weight ratio rather than direct measurements of left ventricular wall thickness via echocardiography; future research could incorporate this parameter for a more precise evaluation of myocardial hypertrophy. The sample size of this study (n = 8 per group, totaling 120 animals) was determined based on the design of previous similar CIA studies, anticipated effect sizes of primary endpoints (such as echocardiographic parameters), and the feasibility of laboratory resources. We acknowledge that the absence of *a priori* power analysis constitutes a limitation of this study. Furthermore, this study utilized a time-series cross-sectional design, in which different animal subgroups were assessed at predetermined disease stages (D0–D112), rather than performing longitudinal tracking of the same cohort throughout the entire disease course. Although this design does not account for the subtle effects of baseline variability among individuals on disease progression trajectories, we enhanced comparability between groups at each time point through strict randomization, which was based on weight-based stratification, and a standardized housing environment. More importantly, the changes in parameters we observed—such as the sustained significant decline in the diastolic function index MVE/A beginning at D84 and the progressive worsening of myocardial fibrosis starting at D56—demonstrated a clear and consistent temporal gradient pattern across all animal subgroups. This consistency across time points strongly indicates that the observed phenomena reflect universal temporal patterns of CIA disease progression, rather than random outcomes resulting from individual variability. However, rigorous longitudinal studies employing implantable probes or more frequent noninvasive monitoring in the future will more accurately delineate the disease trajectory in individual animals. The Sprague-Dawley rat CIA model is a valuable tool for investigating inflammatory arthritis and its related cardiac complications; however, it does not fully replicate the “lipid paradox” seen in patients with rheumatoid arthritis. Additionally, in the absence of dietary or genetic modifications, this model exhibits an inherent resistance to atherosclerosis. This limitation hinders direct translational extrapolation concerning the mechanisms of lipid-driven atherosclerosis. Furthermore, the evaluation of myocardial fibrosis in this study was predominantly qualitative. While Masson staining clearly indicated a progressive increase in collagen deposition, the absence of quantitative data on the area of fibrosis, such as collagen volume fraction, constitutes a significant limitation. Future studies should incorporate morphometric analysis to facilitate more accurate quantification. The experimental design of this study did not allocate sufficient serum samples for subsequent testing, preventing us from directly measuring classic systemic inflammatory markers such as CRP, IL-6, and TNF-α. Consequently, we could not establish a direct quantitative association between circulating inflammation levels and cardiac injury phenotypes. Although the study addressed this limitation by utilizing local joint inflammatory pathology scores as a surrogate marker for systemic inflammatory burden, and indirectly established a mechanistic link between inflammatory activity and cardiac injury through temporal correlation analysis, this approach provides only logical support for the hypothesis that “persistent inflammation drives remote cardiac injury in CIA rats.” Such indirect inference cannot substitute for direct experimental quantitative evidence. Future studies should incorporate the detection of circulating inflammatory markers to elucidate the specific regulatory roles of different pro-inflammatory cytokines in RA-associated cardiac injury. This would validate the hypothesis that “inflammation spillover drives cardiac injury,” thereby enhancing the translational medical significance of this preclinical model in the investigation of RA-associated cardiovascular diseases.

## Data Availability

The original contributions presented in the study are included in the article/supplementary material. Further inquiries can be directed to the corresponding author.
